# Causal Transformer for Learning Embeddings from Structured Medical History Records and Multi-Source Data Integration for Complex Disease Risk Prediction

**DOI:** 10.1007/s12539-025-00749-9

**Published:** 2025-09-17

**Authors:** Zeming Li, Yu Xu, Debajyoti Chowdhury, Hip Fung Yip, Chonghao Wang, Lu Zhang

**Affiliations:** 1 Department of Computer Science, Hong Kong Baptist University, Hong Kong, 999077 China; 2 School of Chinese Medicine, Hong Kong Baptist University, Hong Kong, 999077 China; 3 Institute of Systems Medicine and Health Science, Hong Kong Baptist University, Hong Kong, 999077 China

**Keywords:** Single nucleotide polymorphism, Polygenic risk score, Deep learning, Genome wide association study, Medical history record

## Abstract

**Abstract:**

Traditional disease risk prediction models predominantly rely on statistical algorithms and often focus on genetic factors or a limited set of lifestyle factors to estimate the risk of disease onset. Recently, more comprehensive approaches have emerged that integrate genetic factors with additional lifestyle factors (e.g., alcohol intake) and physical features (e.g., body mass index, age) to increase predictive accuracy. Since the onset of complex diseases is often accompanied by the occurrence of comorbidities, incorporating medical history records is a critical yet underexplored avenue for improving risk prediction. In this study, we propose a novel framework, MIDRP (Multi-source Integration for Disease Risk Prediction), which incorporates genetic variants, lifestyle factors, physical attributes, and medical history records to achieve more robust and accurate predictions. At the heart of our approach lies a causal Transformer architecture, specifically designed to extract and interpret nuanced patterns from medical history records. In the experiments, we compared MIDRP with several baselines, including LDPred2, random forest, multilayer perception, logistic regression, AdaBoost, DiseaseCapsule, EIR, and Med-Bert, on three complex diseases Coronary Artery Disease, Type 2 Diabetes, and Breast Cancer using data from the UK Biobank. Our method achieved state-of-the-art performance, AUROC scores of 0.783, 0.841, and 0.784, respectively, demonstrating its potential in the field of complex disease risk prediction.

**Graphical Abstract:**

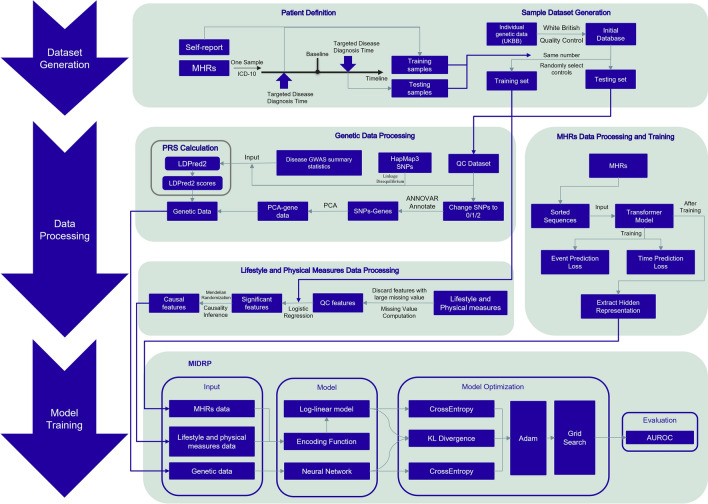

**Supplementary Information:**

The online version contains supplementary material available at 10.1007/s12539-025-00749-9.

## Introduction

Effective and accurate prediction of complex diseases is essential in clinical diagnosis. In recent years, an abundance of big data, artificial intelligence, and robust computational power have allowed clinicians to offer precise and timely treatment plans to their patients. This advancement facilitates rapid stratification of patients into high-risk and low-risk groups, enabling accurate access to primary healthcare services tailored to their specific needs within a broader context. Such clinically relevant computational tools become imperative in primary care and aid clinicians in offering efficient timely treatments to the high-risk group of patients. It minimizes the chances of over-treatment to the low-risk group of patients, optimizing healthcare resources and expenses.

Complex diseases are typically polygenic, involving intricate non-linear interactions between multiple genetic variants and environmental factors [1, 2]. Predicting the risks associated with these diseases presents a significant challenge [3]. Earlier methods, such as simple Neural Networks (NNs), have been explored for disease risk prediction [4]. Yet, these methods often underperform compared to linear models like logistic regression and Lassosum [5, 6], slightly better than random selection, demonstrating its insufficiency to extract significant genetic interaction information in complex diseases. Later, advanced Deep Learning (DL) computational models were increasingly gaining attention in addressing these challenges. In 2022, Luo et al. [7] proposed a method named PCA-Genetic Variant to reduce data dimensionality and extract non-linear information from whole-genome-wide data. They also introduced a DL framework, DiseaseCapsule, to model this PCA-Genetic Variant data, achieving the highest accuracy and F1-score for amyotrophic lateral sclerosis (ALS) from the Dutch cohort of Project MinE.

However, only considering genetic factors may not be sufficient as complex diseases are also affected by other factors such as lifestyles (e.g., alcohol intake), environments, etc. In 2023, Sigurdsson et al. [8] introduced another DL method, EIR. They designed a module, called the locally-connected layer, to extract information from neighboring Single Nucleotide Polymorphisms (SNPs). Similar to Luo et al. [7], EIR performs dimensionality reduction on whole-genome-wide data followed by disease prediction. However, EIR is an end-to-end method that integrates genetic factors with additional non-genetic information. EIR demonstrates a powerful capacity to process non-linear relationships, and its AUROC (area under the receiver operating characteristics curve) outperformed LASSO in Type 1 diabetes (T1D) prediction, as T1D contains significant non-additive genetic information [9].

In addition to the data types mentioned above, Medical History Records (MHR) are another vital data modality. MHR data describe the progressive manifestation of multifactorial diseases and the existence of reported comorbidities for each patient, potentially revealing interactions between different diseases [10, 11]. This data is particularly important for complex chronic diseases, many of which result from a gradual progression of other underlying ailments [12]. For instance, individuals diagnosed with Coronary Artery Disease (CAD) often have comorbidities such as diabetes mellitus, and those with Type 2 Diabetes (T2D) have a 2-4 times higher mortality rate from heart disease compared to those without T2D [13, 14]. Additionally, up to 25% of Americans with T2D also have anemia [14].

MHR-based models have been gradually implemented in recent years. Rasmy et al. [15] introduced Med-Bert, a model designed for sequential data using the BERT framework [16]. It captured underlying disease relationships through masked language modeling tasks and fine-tuned on different downstream tasks by integrating with RNN or GRU head models [17, 18]. Med-Bert model demonstrated good AUROC performance in predicting heart failure and pancreatic cancer using the Cerner Health Facts dataset (version 2017). However, certain aspects of its architecture are unreasonable. For example, the model should focus more on the forward timeline rather than the reverse one, as the sequence of disease occurrences contains crucial information.

In this paper, we introduce a novel framework for robust and accurate prediction of complex disease risk scores. We propose a feature selection component designed to identify lifestyle and physical traits that have a causal relationship with target diseases. By integrating multiple pairs of Genome-Wide Association Studies (GWAS) datasets with two-sample Mendelian Randomization (MR) and synthesizing the results based on meta-analysis [19, 20], we ensure that only the most relevant and causally linked characteristics are selected for further analysis, improving the precision of disease risk prediction. In addition, we introduce Med-Causal-Transformer, a Transformer-based framework trained to extract information from the MHR data by a causal language model (CausalLM) pre-training task. The model is specifically designed to capture nuanced patterns and relationships within the MHR data, providing deeper insight into the underlying mechanisms of the disease and improving the accuracy and interpretability of disease risk predictions. Finally, we propose MIDRP (Multi-source Integration for Disease Risk Prediction), which is built upon our previously proposed model PRSIMD [21]. MIDRP integrates a wide range of data types, including genetic information, lifestyle factors, physical characteristics (e.g., BMI, age), and MHR data by posterior regularization [22, 23]. In our experiments, MIDRP showed state-of-the-art performance in CAD, T2D, and Breast Cancer (BC) datasets from UK Biobank (UKBB) [24], achieving AUROC scores of 0.783, 0.841, and 0.784, respectively.

## Methods

### Processing UKBB Genotype Data

The genotype data was processed by PLINK1.9 to select high-quality samples and SNPs [25]. The initial dataset includes 337,536 samples that meet the following criteria: (1) self-reported ancestry is White British; (2) samples have less than 10% SNPs with missing genotypes; (3) samples are not outliers or related samples that are excluded from principal component calculations; (4) samples never withdraw their data. In addition, 287,679 SNPs were removed for failing to meet at least one of the following criteria: (i) their minor allele frequencies are lower than 1%; (ii) their *P*-values of Hardy Weinberg equilibrium are lower than 1×10$^{-6}$; (iii) they show genotype missing rates that exceed 10%.

### Patient Definition and Dataset Generation for Three Diseases with External Summary Statistics

We define patient labels based on self-reports disease field in the UKBB and ICD-10 codes in first-level diagnosis in MHR, following the criteria described in [4]. To enhance the rationality and reliability of the following method evaluation, we apply a strict limitation for splitting the data into training and testing sets. We first set a baseline (time of attending the assessment center) for each patient. Then, if the earliest diagnosis time of the target disease in the patients’ MHR is earlier than the baseline, the patients are assigned to the training set; otherwise, the patients are assigned to the testing set. In addition, we assign individuals who self-report having the target disease to the training set. Furthermore, we conduct a control pool for the selected disease (samples never diagnosed with the corresponding disease), and randomly select the same number of controls as the training and testing samples. We finally integrate them together as the training and testing sets. The participants in this section are restricted to the above section’s sample set.

### Processing UKBB Lifestyles and Physical Features

The dataset is initiated with White British samples (442,591) for lifestyles and physical features. We perform missing value computation for these features and adopt the most frequency and median data processing strategy for categorical and numerical features, respectively. A missing rate greater than 5% can significantly impact the computation; therefore, features with more than 22,129 missing values (5% of 442,591) are filtered out. Afterward, we generate a candidate feature list for each disease by applying logistic regression to fit the training set, and the features are excluded if *P*-values > 0.05.

### Processing UKBB Medical History Data for Med-Causal-Transformer Model

The participants are eliminated from the initial dataset if they do not have MHR data and their medical history records number is less than 2. We only use the first-level diagnosis records, specifically focusing on the first three digits of the ICD-10 codes, which amount to a total of 1481 3-digit codes within our dataset. To train the Med-Causal-Transformer model, we convert every 3-digit ICD-10 code into a distinct numerical representation and organize the patient’s MHR by sorting their records according to the admission time.

### Rationale for Selecting CAD, T2D and BC in UKBB

Our study requires the integration of four data modalities: lifestyle factors, physical features, medical history records, and genetic data, and these datasets are linked by the EID field. However, the sample sizes for each dataset differ due to the fact that they were not collected simultaneously by UKBB. In addition, during the process of screening samples and splitting the data into training and testing sets, we apply a series of criteria and limitations (refer to Sects. 2.1, 2.2, 2.3 and 2.6) to ensure the rationality and reliability of our experiments. These criteria inevitably reduce the number of available samples. Finally, to ensure the robustness of the study and prove that our proposed methods can be extended to different scenarios, we select CAD (15,426 for training, 6436 for testing), T2D (4666 for training, 990 for testing), and BC (16,896 for training, 7104 for testing) due to their sufficient training and testing sample size.

### GWAS Processing in MR Analysis and Causal Features Selection

To identify causal relationships between lifestyle and physical features and target diseases, we integrate two-sample MR with a meta-analysis approach. Two-sample MR is a method that assesses causality by leveraging the statistical correlation between genetic variants and an exposure variable, as well as between genetic variants and an outcome variable, and strictly relies on three core assumptions [19]: (1) the genetic variants must be strongly correlated with the exposure variable; (2) the genetic variants must be independent of confounding factors; (3) the genetic variants should influence the outcome variable solely through the exposure variable. In our study, lifestyle and physical features serve as the exposure variables, while the diseases represent the outcome variables.

GWAS used in our MR analysis are sourced from the OpenGWAS Project [26–28]. To ensure the robustness of our instrumental variables, we apply stringent filtering criteria to the GWAS data. For each exposure variable, we exclude SNPs [29] if: (1) their association *P*-values exceed $1\times 10^{-5}$, or (2) their minor allele frequencies are below 0.01. This filtering process helps to construct instrumental variables that are less susceptible to confounding factors and have a strong association with the target exposure variables. We further minimize the risk of horizontal pleiotropy where a genetic variant influences multiple traits independently of the exposure variable using PhenoScanner [30], an online database that checks the associations between SNPs and various traits. SNPs with a *P*-value smaller than $5\times 10^{-8}$ are identified as having extremely significant relationships with other traits and are considered potential confounders. These SNPs are subsequently excluded from our analysis. Additionally, we perform linkage disequilibrium (LD) pruning on the remaining SNPs using the 1000 Genomes Reference Panel (clump distance $=$ 10,000, clump $r^{2}$ $=$ 0.001) to reduce the likelihood of vertical pleiotropy, ensuring that the residual SNPs are suitable as instrumental variables for each exposure variable [31].

For each disease, we select two distinct GWAS datasets for the same outcome variable and combine each with one exposure GWAS, resulting in two data pairs (each pair consisting of one exposure GWAS and one outcome GWAS). We then apply the Inverse Variance-Weighted (IVW) method to these data pairs based on the selected instrumental variables, generating two MR results [32]. A meta-analysis is subsequently conducted on these two MR results to produce a consistent and robust causal inference. We consider an exposure variable to be causally related to an outcome variable if it meets all of the following criteria: (1) the meta-analysis fixed-effect *P*-value is lower than 0.05; (2) the meta-analysis $I^{2}$ statistic is lower than 0.05, indicating low heterogeneity; (3) the meta-analysis heterogeneity *P*-value exceeds 0.05; (4) the confidence interval for the fixed-effect model either has its upper bound below 0 or its lower bound above 0.

In the experiment, we initially evaluated 100, 87, and 61 candidate features for CAD, T2D, and BC, respectively. Using PhenoScanner, we excluded a total of 30,051 SNPs across all MR analyses. These SNPs show significant associations with potential confounders unrelated to the target exposures (see **Supplementary Data A and B**). Following the meta-analysis, we retain the features with robust causal relationships, which are 85 for CAD, 59 for T2D, and 16 for BC (see **Supplementary Data C**).

### Med-Causal-Transformer Introduction

#### Input Embedding

1 $$\begin{aligned} {[}{\boldsymbol{{z}}}(t_{i})]_{j}={\left\{ \begin{array}{ll} \cos (t_{i}/10000^{\frac{j-1}{M} } ),\quad \text {if}\ j\ \text {is odd} \\ \sin (t_{i}/10000^{\frac{j}{M} } ),\quad \text {if}\ j\ \text {is even}\end{array}\right. } \end{aligned}$$

The position encoding in the traditional Transformer model is modified into temporal encoding [33, 34], as shown in Eq. (1), for the self or mutually excited event sequence data. In Eq. (1), $t_{i}$ is the time, *i* is the position of $t_{i}$ in the sequence, *j* represents the position of the temporal vector, *M* is the dimension of encoding, ${\boldsymbol{{z}}}(t_{i}) \in \mathbb {R}^{M}$. For event type embedding, we generate a learnable matrix ${\boldsymbol{{U}}} \in \mathbb {R}^{M\times K}$ where *M* is the embedding dimension, and *K* is the number of event types. We set an event type ${\boldsymbol{{k}}}_{i} \in \mathbb {R}^{K}$ is a one-hot encoding with *K* dimension, so its event type embedding is ${\boldsymbol{{U}}}{\boldsymbol{{k}}}_{i}$. For a sequence ${\boldsymbol{{S}}} = [{{\boldsymbol{{t}}}_{i}, {\boldsymbol{{k}}}_{i}}]$, the embedding could be defined by

2 $$\begin{aligned} {\boldsymbol{{X}}} = ({\boldsymbol{{UY}}}+{\boldsymbol{{Z}}})^{\mathrm{T}}\end{aligned}$$

where ${\boldsymbol{Y}} = [{\boldsymbol{k}}_{1},{\boldsymbol{k}}_{2},{\boldsymbol{k}}_{3},\ldots ,{\boldsymbol{k}}_{L}] \in \mathbb {R}^{K\times L}$ is a collection of event types, *L* is the sequence length, ${\boldsymbol{UY}} \in \mathbb {R}^{M\times L}, {\boldsymbol{Z}} =[{\boldsymbol{z}}(t_{1}),{\boldsymbol{z}}(t_{2}),{\boldsymbol{z}}(t_{3}), \ldots ,{\boldsymbol{z}}(t_{L})] \in \mathbb {R}^{M\times L}$ is a collection temporal encoding of a sequence. It is worth noticing that ${\boldsymbol{UY}}$ has the same dimension as *Z*, ${\boldsymbol{X}} \in \mathbb {R}^{L\times M}$, and each row of ${\boldsymbol{X}}$ corresponds to the addition between the specific event type embedding and the temporal encoding. Then, ${\boldsymbol{X}}$ will be set as inputted into the Med-Causal-Transformer model.

#### The Architecture of Med-Causal-Transformer Model

***Multi-head Masked Attention Network*** After passing through the embedding layers, the event sequence is transformed into ${\boldsymbol{X}}$, and then we feed ${\boldsymbol{X}}$ into the Med-Causal-Transformer model. The attention matrix ${\boldsymbol{S}}$ could be specified by

3 $$\begin{aligned} {\boldsymbol{S}}= & \text {Softmax}(\frac{{\boldsymbol{QK}}^{\text {T}}}{\sqrt{M_{{\boldsymbol{K}}}}}){\boldsymbol{V}}) \end{aligned}$$

4 $$\begin{aligned} {\boldsymbol{Q}}= & {\boldsymbol{XW}}^{{\boldsymbol{Q}}}, {\boldsymbol{K}}={\boldsymbol{XW}}^{{\boldsymbol{K}}}, {\boldsymbol{V}}={\boldsymbol{XW}}^{{\boldsymbol{V}}} \end{aligned}$$

where ${\boldsymbol{Q}}$, ${\boldsymbol{K}}$, and ${\boldsymbol{V}}$ are respectively query, key, and value. ${\boldsymbol{W}}^{{\boldsymbol{Q}}}\in \mathbb {R}^{M\times M_{{\boldsymbol{Q}}}}$, ${\boldsymbol{W}}^{{\boldsymbol{K}}}\in \mathbb {R}^{M\times M_{{\boldsymbol{K}}}}$, and ${\boldsymbol{W}}^{{\boldsymbol{V}}}\in \mathbb {R}^{M\times M_{{\boldsymbol{V}}}}$. In reality, we always use the multi-attention module, ${\boldsymbol{W}}^{\text {multi}}=\left\{ {\boldsymbol{W}}^{Q}, {\boldsymbol{W}}^{K}, {\boldsymbol{W}}^{V} \right\} _{h=1}^{H}$ $\in \mathbb {R}^{HM\times M_{{\boldsymbol{Q}}}}$, to improve the models ability. Several attention matrices ${\boldsymbol{A}}_{1}$,${\boldsymbol{A}}_{2}$,...,${\boldsymbol{A}}_{H}$ will be generated from different sets of ${\boldsymbol{W}}^{\text {multi}}$, and the multi-attention module s output could be specified by ${\boldsymbol{S}}=\left[ {\boldsymbol{A}}_{1}, {\boldsymbol{A}}_{2}, \ldots , {\boldsymbol{A}}_{H} \right] {\boldsymbol{V}}^{\text {multi}}$, where ${\boldsymbol{V}}^{\text {multi}} \in \mathbb {R}^{HM_{{\boldsymbol{V}}}\times L}$ and *L* is the sequence length.

**Fig. 1:**
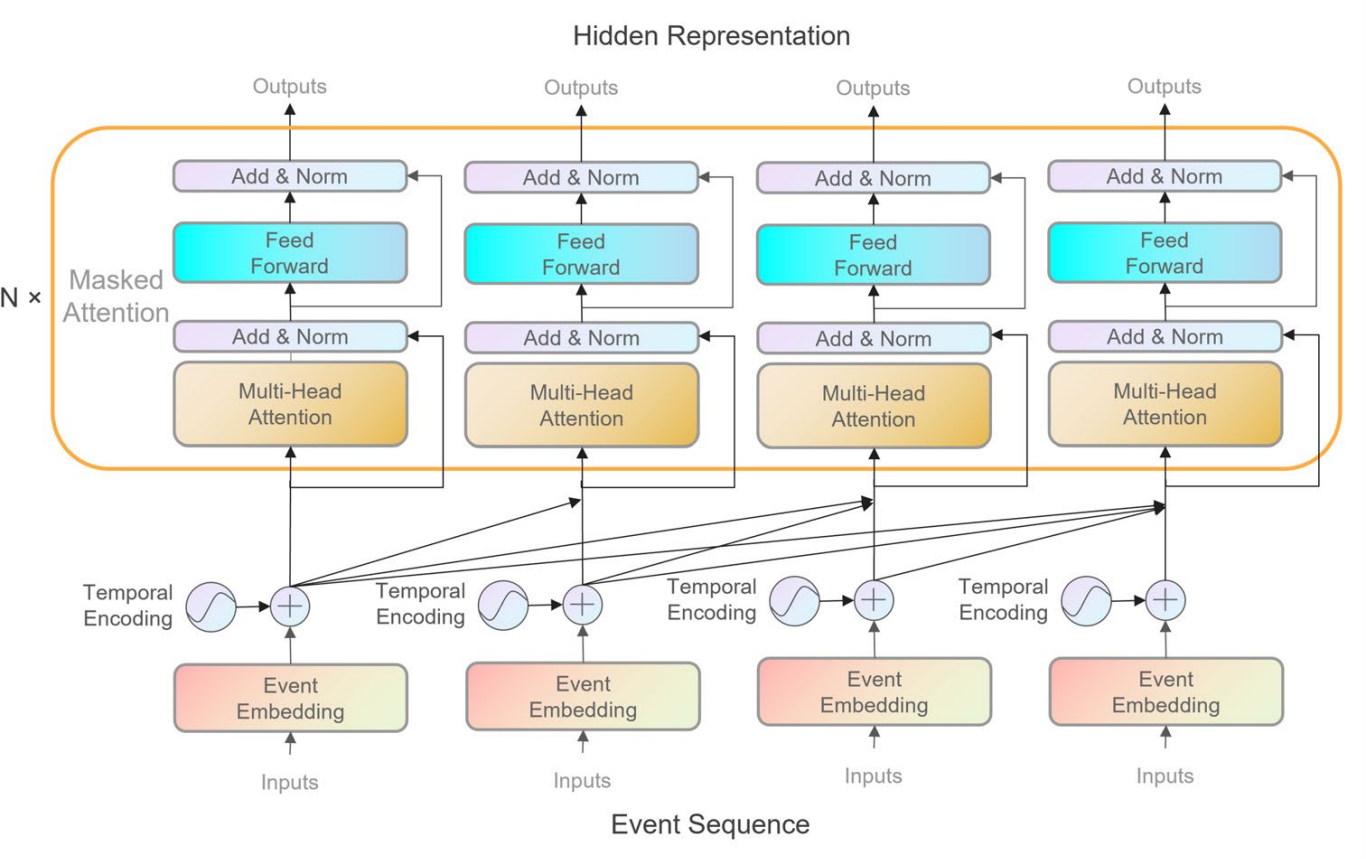
Architecture of the Med-Causal-Transformer model. For an input sequence, firstly, go through embedding layers and then pass through *n* masked attention models. The output hidden representation for each event contains MHR information that precedes it

Since the Med-Causal-Transformer model must obey a guideline, the $j_{\text {th}}$ event of a sequence can only observe the past. We use the multi-head masked attention network (Fig. 1) similar to the multi-head attention network. We set ${\boldsymbol{E}} = {\boldsymbol{QK}}^\mathrm{T}$, where ${\boldsymbol{E}}\in \mathbb {R}^{L\times L}$. Then, we create an upper triangular matrix filled with 1 to specify which column element should be masked for each row. After that, we set the masked elements to $-\infty$ in ${\boldsymbol{E}}$. This operation could make the attention weights corresponding to the masked elements equal to 0 after passing through the Softmax function. Then, the multi-head masked attention module s output ${\boldsymbol{S}}$ will go through the feed-forward network and generate the hidden representation. The formula is defined as follows:

5 $$\begin{aligned} {\boldsymbol{H}}=\text {ReLU}({\boldsymbol{SW}}_{1}^{\text {FC}}+{\boldsymbol{b}}_{1}){\boldsymbol{W}}_{2}^{\text {FC}}+{\boldsymbol{b}}_{2} \end{aligned}$$

where ${\boldsymbol{W}}_{1}^{\text {FC}} \in \mathbb {R}^{M \times M_{H}}$ and ${\boldsymbol{W}}_{2}^{\text {FC}} \in \mathbb {R}^{M_{H} \times M}$ are the weight matrices for the two layers of the feed-forward network; ${\boldsymbol{b}}_{1} \in \mathbb {R}^{M_{H}}$ and ${\boldsymbol{b}}_{2} \in \mathbb {R}^{M}$ are the corresponding bias vectors. Here, $M_{H}$ denotes the dimensionality of the hidden state in the feed-forward network. Additionally, ${\boldsymbol{S}} = [{\boldsymbol{s}}_{1}, {\boldsymbol{s}}_{2}, \ldots , {\boldsymbol{s}}_{L}] \in \mathbb {R}^{L \times M}$ is a matrix where each row ${\boldsymbol{s}}_{i}$ represents the hidden representation of a specific event. In practice, we set the number of layers to 4, the number of heads to 4, the dimension of ***K*** and ***Q*** to 128, and the inner dimension of the feed-forward network to 256.

### Med-Causal-Transformer Objective Function and Training

After a sequence event passing through the multi-head masked attention models, we obtain a hidden representation ${\boldsymbol{H}}$. Then, we separately use a dense network separately to predict the next event and the occurrence time, which can be represented as follows:

6 $$\begin{aligned} \hat{t}_{i+1}= & {\boldsymbol{W}}_{\text {time}}^{\text {FC}}h({\boldsymbol{s}}_{i}) \end{aligned}$$

7 $$\begin{aligned} \hat{{\boldsymbol{k}}}_{i+1}= & \underset{k}{\text {argmax}}(\text {Softmax}({\boldsymbol{W}}_{\text {event}}^{\text {FC}}h({\boldsymbol{s}}_{i}))) \end{aligned}$$

where $${\boldsymbol{W}}_{\text {time}}^{\text {FC}}\in \mathbb {R}^{M}$$ and $${\boldsymbol{W}}_{\text {event}}^{\text {FC}}\in \mathbb {R}^{M\times K}$$.

In practice, we respectively calculate the mean square error and the cross entropy loss for time prediction and event prediction tasks, and the equations are defined as follows:

8 $$\begin{aligned} \mathcal {L}_{\text {time}}(S_{n})= & {\textstyle \sum _{i=2}^{I_{n}}}(t_{i}-\hat{t}_{i})^{2} \end{aligned}$$

9 $$\begin{aligned} \mathcal {L}_{\text {event}}(S_{n})= & {\textstyle \sum _{i=2}^{I_{n}}}-c_{i}\text {log}(\hat{p}_{i}) \end{aligned}$$

where $t_{i}$ is the true time, $\hat{t}_{i}$ is the predicted time, $c_{i}$ is the true event label, and $\hat{p}_{i}$ is the event predicted probability.

Lastly, the overall objective loss for training the Med-Causal-Transformer model is

10 $$\begin{aligned} \min \sum _{n=1}^{N} \alpha _{\text {event}}\mathcal {L}_{\text {event}}(S_{n}) + \alpha _{\text {time}}\mathcal {L}_{\text {time}}(S_{n}) \end{aligned}$$

where $\alpha _{\text {event}}$ and $\alpha _{\text {time}}$ are two hyperparameters adjusting $\mathcal {L}_{\text {event}}(S_{n})$ and $\mathcal {L}_{\text {time}}(S_{n})$.

Here, we discuss some key advantages of the Med-Causal-Transformer. Throughout the entire training process, we deal with the aforementioned issues: (1) samples with diagnostic data of varying lengths can be processed after completion and clipping strategies; (2) the model benefits from the masked attention module, which prevents the model from using future data points that would invalidate the predictive process; (3) profiting from the CausalLM training strategy, potential MHR information can be extracted for downstream disease prediction tasks without fintune.

### Attention Pattern Visualization

In this study, we use BertViz [35], a Python library, to visualize attention patterns within the Med-Causal-Transformer model. This tool allows for the visualization of one or more attention heads within the same layer, each represented by a different color, with color depth indicating the strength of the correlation.

### MIDRP

#### Modeling Genetic Data

MIDRP uses logistic regression to transform the polygenic risk scores (PRSs) for each disease, which were calculated by PRS calculation methods such as P+T, LDPred2, DiseaseCapsule, EIR and so on, into a probability distribution [7, 8, 36, 37]. The equations are specified by

11 $$\begin{aligned} & L_{\text {genetic}}(y=1|x_{s};\theta )=w_{s}x_{s}+b_{s} \end{aligned}$$

12 $$\begin{aligned} & P(y=1|x_{s};\theta )=(1+\text {e}^{-L_{\text {genetic}}(y=1|x_{s};\theta )})^{-1} \end{aligned}$$

13 $$\begin{aligned} & P(y=0|x_{s};\theta )=1-P(y=1|x_{s};\theta ) \end{aligned}$$

where $L_{\text {genetic}}(\cdot )$ is the logit function for genetic data, $y_{i}$ is the disease label, $y_{i} \in \left\{ 0, 1 \right\}$, $x_{s}$ is the PRS score, $w_{s}$ is the weight, and $b_{s}$ is the bias.

#### Modeling Non-genetic Data

**Fig. 2:**
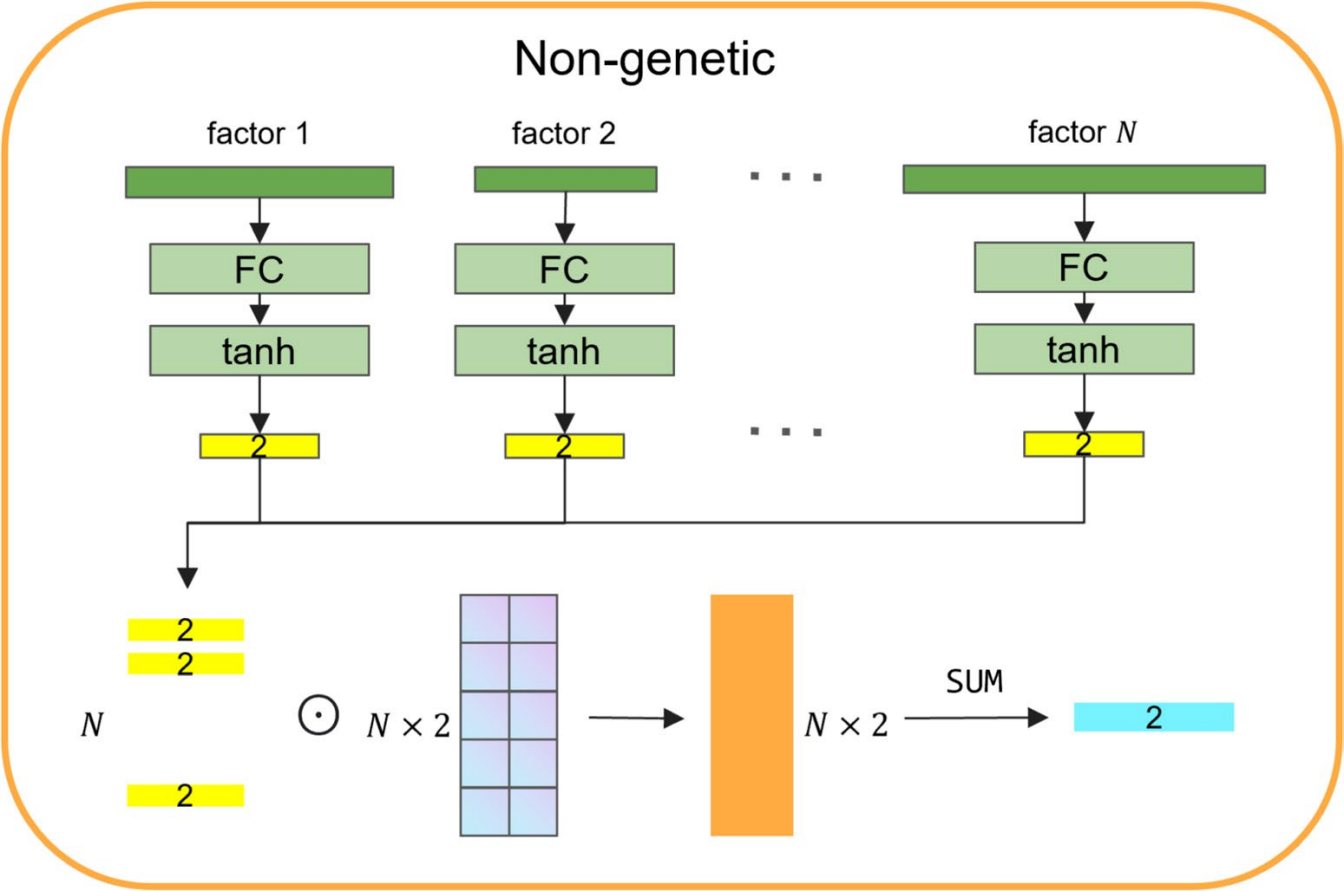
The architecture of modeling non-genetic data. For one sample, each non-genetic factor will be fed into an FC network followed by a tanh activation function and transformed into a vector with a shape of 2. Then get a matrix with the shape of [N, 2] by concatenating all factors output vectors. Finally, the matrix will be weighted by a learnable matrix with the same dimension and perform a sum action in the second dimensionality

The process of modeling non-genetic data is shown in Fig. 2. Firstly, we set a function $\phi$ for each factor, and the function is defined by $\phi ({\boldsymbol{x}}_{j},y;{\boldsymbol{w}}_{j})= \text {tanh}({\boldsymbol{w}}_{j}{\boldsymbol{x}}_{j})$, where ${\boldsymbol{x}}_{j}$ is the $j_{\text {th}}$ non-genetic factor, ${\boldsymbol{x}}_{j} \in \mathbb {R}^{M}$, ${\boldsymbol{w}}_{j}$ is the weight for ${\boldsymbol{x}}_{j}$, and ${\boldsymbol{w}}_{j} \in \mathbb {R}^{M\times 2}$. It is worth noticing that different factors have different input dimensions.

After getting $\boldsymbol{\phi }^{\text {all}} = \left\{ \boldsymbol{\phi }_{1}, \boldsymbol{\phi }_{2}, \ldots , \boldsymbol{\phi }_{N}  \right\}\boldsymbol{\phi }^\mathrm{all}$, $\boldsymbol{\phi }^{all} \in \mathbb {R}^{N\times 2}$, we create a weight matrix $\boldsymbol{\Gamma } \in \mathbb {R}^{N\times 2}$, each row corresponds to each factor, and the output of this part could be formulated by

$$\begin{aligned} L_{\text {non-genetic}}(y|{\boldsymbol{X}};\boldsymbol{\Gamma },{\boldsymbol{W}})=\text {Sum}(\Gamma \odot \text {Concat}_{j=1}^{N}(\phi ({\boldsymbol{x}}_{j},y;{\boldsymbol{w}}_{j}))) \end{aligned}$$

where $\odot$ represents the Hadamard product.

Then we transform the $L_{\text {non-genetic}}(y|{\boldsymbol{X}};\boldsymbol{\Gamma },{\boldsymbol{W}})$ into a probability distribution; the equation is specified by

$$\begin{aligned} Q(y|{\boldsymbol{X}};\boldsymbol{\Gamma },{\boldsymbol{W}})=\frac{\text {exp}(L_{\text {non-genetic}}(y|{\boldsymbol{X}};\boldsymbol{\Gamma },{\boldsymbol{W}}))}{ {\textstyle \sum _{y \in \left\{ 0,1 \right\} } \text {exp}(\text {non-genetic}}(y|{\boldsymbol{X}};\boldsymbol{\Gamma },{\boldsymbol{W}})} \end{aligned}$$

We also adopt the cross entropy loss to calculate the objective loss for the non-genetic part. In practice, we additionally adopt posterior regularization technology, using $\text {KL}$ divergence to make probability distribution *P* close to *Q*. Suppose we have *N* samples, then the equation of KL divergence loss could be defined as follows:

$$\begin{aligned} \mathcal {L}_{\text {KL}}(\theta ,\boldsymbol{\Gamma },{\boldsymbol{W}})=\frac{1}{K}\sum _{i=1}^{K} \text {KL}(Q(y^{(i)}|X^{(i)};\boldsymbol{\Gamma },{\boldsymbol{W}})\parallel P(y^{(i)}|x_{s}^{(i)};\theta )) \end{aligned}$$

The overall objective function is defined by

$$\begin{aligned} \mathcal {L}(\theta ,\boldsymbol{\Gamma },{\boldsymbol{W}})=\mathcal {L}_{P}(\theta )+\alpha \mathcal {L}_{\text {KL}}(\theta ,\boldsymbol{\Gamma },{\boldsymbol{W}})+\beta \mathcal {L}_{Q}(\boldsymbol{\Gamma },{\boldsymbol{W}}) \end{aligned}$$

where $\mathcal {L}_{P}$ and $\mathcal {L}_{Q}$ are respectively genetic and non-genetic loss; $\alpha$ and $\beta$ are two hyperparameters used to tune $\mathcal {L}_{\text {KL}}$ and $\mathcal {L}_{Q}$. Lastly, the equation of the risk score of MIDRP is specified by

$$\begin{aligned} \textit{R} = L_{\text {genetic}}(y=1|x_{s};\theta )+L_{\text {non-gentic}}(y=1|{\boldsymbol{X}};\boldsymbol{\Gamma }, {\boldsymbol{W}}). \end{aligned}$$

### Comparison Methods and Input Specifications

We select eight baseline approaches that represent four distinct computational paradigms for disease risk prediction. The input data specifications were standardized across all selected models (see Supplementary Table 1). The genetic variant-based model LDPred2 utilizes genotyped SNPs, coded as 0/1/2 for minor allele counts, and selects high-quality variants based on HapMap3. The PCA genetic variant-based models, including DiseaseCapsule, MLP and conventional machine learning methods such as RF, LR, and AdaBoost, use principal components derived from gene-centric SNP aggregation. For CAD, T2D, and BC, SNPs are mapped to 9171, 9057, and 8952 genes respectively, and principal component analysis is applied to extract non-linear information within each gene region. The multimodal integration model EIR employs a hybrid input structure that includes genotyped SNPs filtered at a significance level of *P*<0.05, as well as lifestyle factors. The medical history-based model Med-BERT processes ICD-10 codes as sequential inputs, with a vocabulary of 1,481 unique 3-digit ICD-10 categories.

## Results

**Fig. 3:**
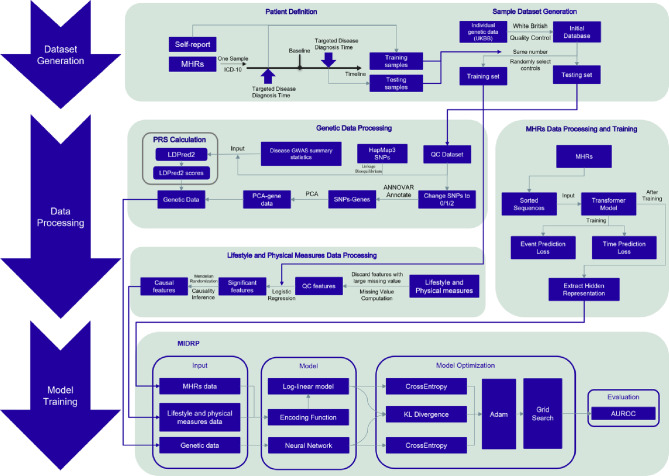
Workflow for training MIDRP. The workflow consists of three modules. **a** Dataset generation module. **b** Data processing module. **c** Model training module

We develop a complex disease prediction framework, MIDRP, which integrates multiple data modalities and achieve state-of-the-art performance on three disease datasets: CAD, T2D, and BC. These datasets are sourced from UKBB. For an overview of the data processing and model training procedures, please refer to Fig. 3. Detailed methodological information was provided in Sect. 2.

### Workflow

The workflow of the proposed framework is organized into 4 steps: (1) Patient Definition, as detailed in Sect. 2.2: the first step involves defining the patient cohort and establishing a control pool for random sampling of controls. (2) Auto-Selection of Causal Features, as described in Sect. 2.6: in the second step, we collect open-source GWAS data. After employing basic QC, we utilize MR to filter features that demonstrate a causal relationship with the target disorder. (3) Med-Causal-Transformer Training, as explained in Sects. 2.4 and 2.8: in this step, MHR is transformed into numerical data and serves as input for the model. This training is based on the CausalLM pretraining task, which is crucial for extracting MHR information. Additionally, in testing procedure, we set different guidelines for extracting MHR information for cases and controls. For cases: (i) if the first diagnosis of the targeted disease occurs at time $t_{j}$, we extract the hidden representation of $t_{j-1}$ as its MHR information; (ii) if the first diagnosis of the targeted disease occurs at time $t_{0}$, we assign a zero vector as its MHR information. For controls: we extract the hidden representation of the last diagnosis event as MHR information. (4) MIDRP Training and Evaluation, as discussed in Sect. 2.10: in the final step, all previously gathered data are used to train the MIDRP model.

In the experiments, to assess the effectiveness of MHR in improving predictive accuracy for complex diseases, we compared the performance of MIDRP across different types of input data. In addition, we evaluated MIDRP against several baseline models (see **Supplementary Table ** 1). These models include LDPred2, RF, MLP, LR, AdaBoost, DiseaseCapsule, EIR, and Med-BERT (BI-GRU). For the RF, MLP, LR and Adaboost models, we use default parameters from Scikit-learn (version 1.3.0) [38]. For other models, LDPred2, PRS-CS, EIR, and Med-BERT, we apply their GitHub codebases [8, 15, 37, 39]. All computations were executed on a server with the following specifications: AMD EPYC 7513 (2 S/32C) @2.1 GHz/1 TB RAM, 2 sets of Nvidia Ampere A100 80 GB, Oracle Linux 8.8 and CUDA 11.8.

### Evaluating the Effectiveness of Extracted MHR Information

**Table 1:** Classification results of single modality and multi-modal data input

Task	Modality	Accuracy	Precision	Recall	F1 score
**CAD**	Only involving MHR information$^{1}$	0.690	0.728	0.606	0.662
	Without MHR information$^{2}$	0.650	0.629	**0**.**731**	0.676
	With MHR information$^{3}$	**0**.**716**	**0**.**730**	0.685	**0**.**706**
**T2D**	Only involving MHR information$^{1}$	0.665	0.676	0.632	0.653
	Without MHR information$^2$	0.754	0.741	**0**.**780**	0.760
	With MHR information$^{3}$	**0**.**773**	**0**.**800**	0.733	**0**.**763**
**BC**	Only involving MHR information$^{1}$	0.706	0.824	0.523	0.640
	Without MHR information$^{2}$	0.589	0.609	0.497	0.547
	With MHR information$^{3}$	**0**.**719**	**0**.**785**	**0**.**602**	**0**.**682**

$^{1}$MLP, $^{2}$PRSIMD, $^{3}$MIDRP

**Fig. 4:**
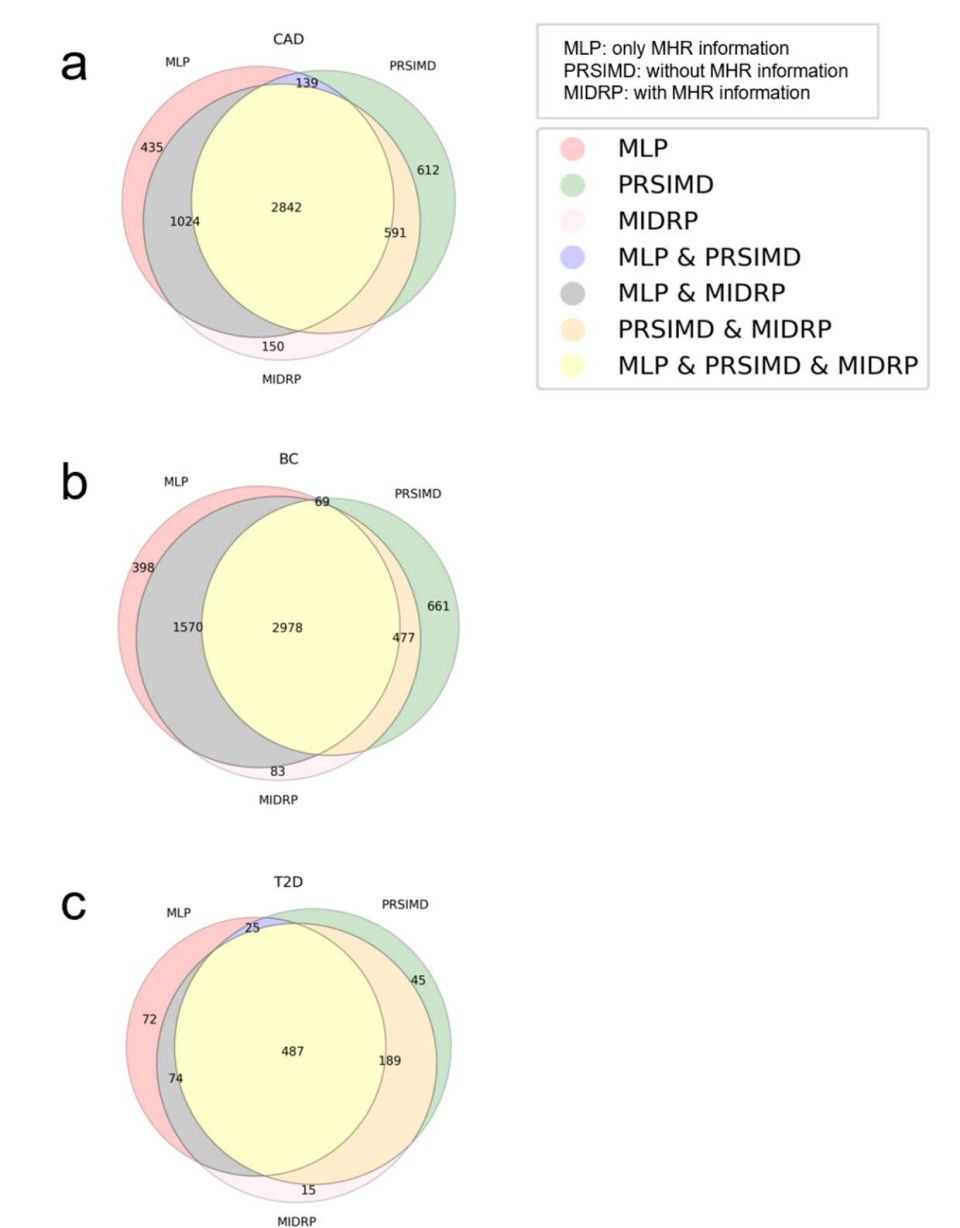
Venn diagrams of correct prediction number for disease predictions using three different data types. The diagrams involve MLP (with MHR information), PRSIMD (with LDPred2 result, lifestyles, and physical features), and MIDRP (with LDPred2 result, lifestyles, and physical features and MHR information). **a** Prediction results of CAD testset. **b** Prediction results of T2D testset. **c** Prediction results of BC testset

The effectiveness of extracted MHR information is justified with the presented classification results of MLP (only involving MHR information) and PRSIMD, as shown in Table 1. We find that MLP and PRSIMD win or lose to each other in different disease prediction tasks, and both could achieve an accuracy of around 0.6 or above in predicting diseases. Each model has a substantial portion of unique correct predictions apart from the overlapping accurate predictions, see Fig. 4. With the incorporation of MHR information, the prediction performance of MIDRP improves not only in terms of accuracy measures but also significantly in Recall and F1 scores, and the correctly predicted samples encompass not only the shared portions from MLP and PRSIMD but also their unique segments. This observation further verifies the effectiveness of the extracted MHR information.

### Comparison of MIDRP to Different State-of-the-art Models

**Fig. 5:**
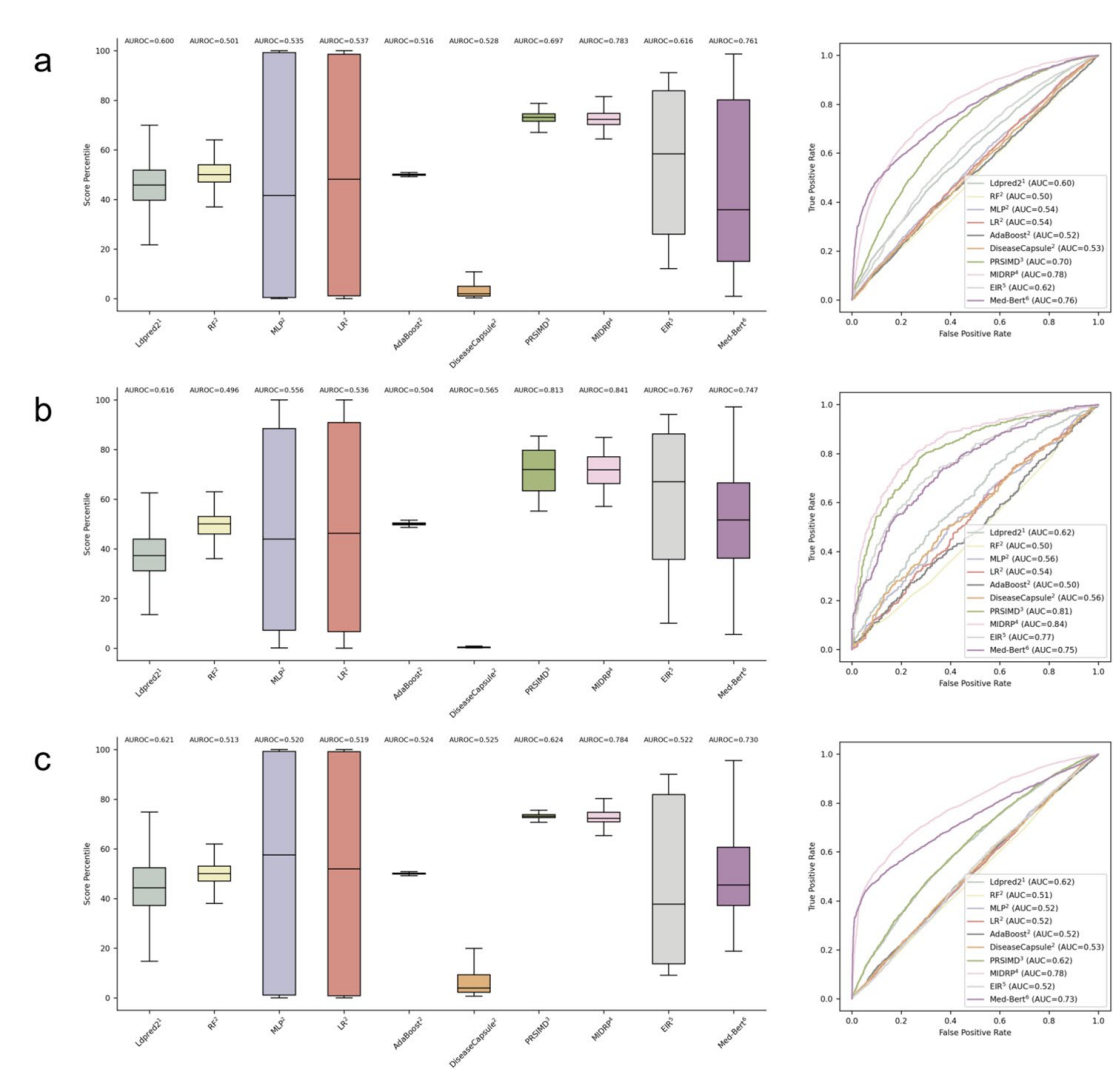
The predictive performance on CAD, T2D, and BC. The boxplots of the predicted score percentiles and ROC curves of CAD (**a**), T2D (**b**), and BC (**c**) on ten methods. $^{1}$: involving genetic variant; $^{2}$: involving PCA-Genetic Variant; $^{3}$: involving LDPred2 result, lifestyles, and physical features; $^{4}$: involving LDPred2 result, lifestyles, physical features, and MHR; $^{5}$: involving genetic variant (*P*-value < 0.05), lifestyle, and physical features; $^{6}$: involving MHR

In this experiment, we compared MIDRP with several baseline methods and observed that MIDRP reached state-of-the-art performance (see Fig. 5). Our previously proposed PRSIMD model demonstrates superior performance compared to several baseline methods that utilize genetic variants, lifestyles, or physical characteristics, in CAD and T2D datasets. PRSIMD only narrowly lag behind Med-BERT with MHR data in the BC dataset. With the introduction of MIDRP, we observed a performance improvement in these three datasets. MIDRP achieves an 8.6% improvement in the CAD task and a 2.8% improvement in the T2D task compared to the PRSIMD model. Furthermore, MIDRP outperforms Med-BERT in the BC task, showing a 5.4% improvement. Furthermore, the predictive score distributions of MIDRP, as depicted in the boxplots in Fig. 5, are relatively stable compared to other models (CAD $Q_{1}$=70.25, $Q_{3}$=74.78; T2D $Q_{1}$=66.30, $Q_{3}$=77.13; BC $Q_{1}$=70.87, $Q_{3}$=74.70). Overall, these findings highlight the superior effectiveness and its potential to outperform existing models.

Since MIDRP is designed to incorporate different genetic risk scores derived from diverse methodologies, we aim to substantiate the state-of-the-art capacity of MIDRP further by conducting an ablation study, ensuring a fair comparison condition (Table 2). The results show that the AUROC performance for DiseaseCapsule and EIR increases significantly: (i) for CAD, from 0.528 to 0.770 (DiseaseCapsule) and from 0.616 to 0.736 (EIR); (ii) for T2D, from 0.565 to 0.839 (DiseaseCapsule) and from 0.767 to 0.833 (EIR); (iii) for BC, from 0.525 to 0.744 (DiseaseCapsule) and from 0.522 to 0.660 (EIR). Overall, these results demonstrate two conclusions: (1) The performance improvements are achieved by applying MIDRP with the output scores generated by the established approaches. (2) The robustness and generalizability of MIDRP are confirmed. While these results do not exceed that of the approach utilizing LDPred2 results as input, they remain superior to most alternative methods.

**Table 2:** Ablation experiment of different genetic risk scores input

Methods	AUROC		
	CAD	T2D	BC
DiseaseCapsule	0.528	0.565	0.525
MIDRP$^{1}$	0.770	0.839	0.744
EIR	0.616	0.767	0.522
MIDRP$^{2}$	0.736	0.833	0.660

$^{1}$ Involving DiseaseCapsule result. $^{2}$ Involving EIR result

### Med-Causal-Transformer’s Capability in Extracting Disease-disease Interaction

**Fig. 6:**
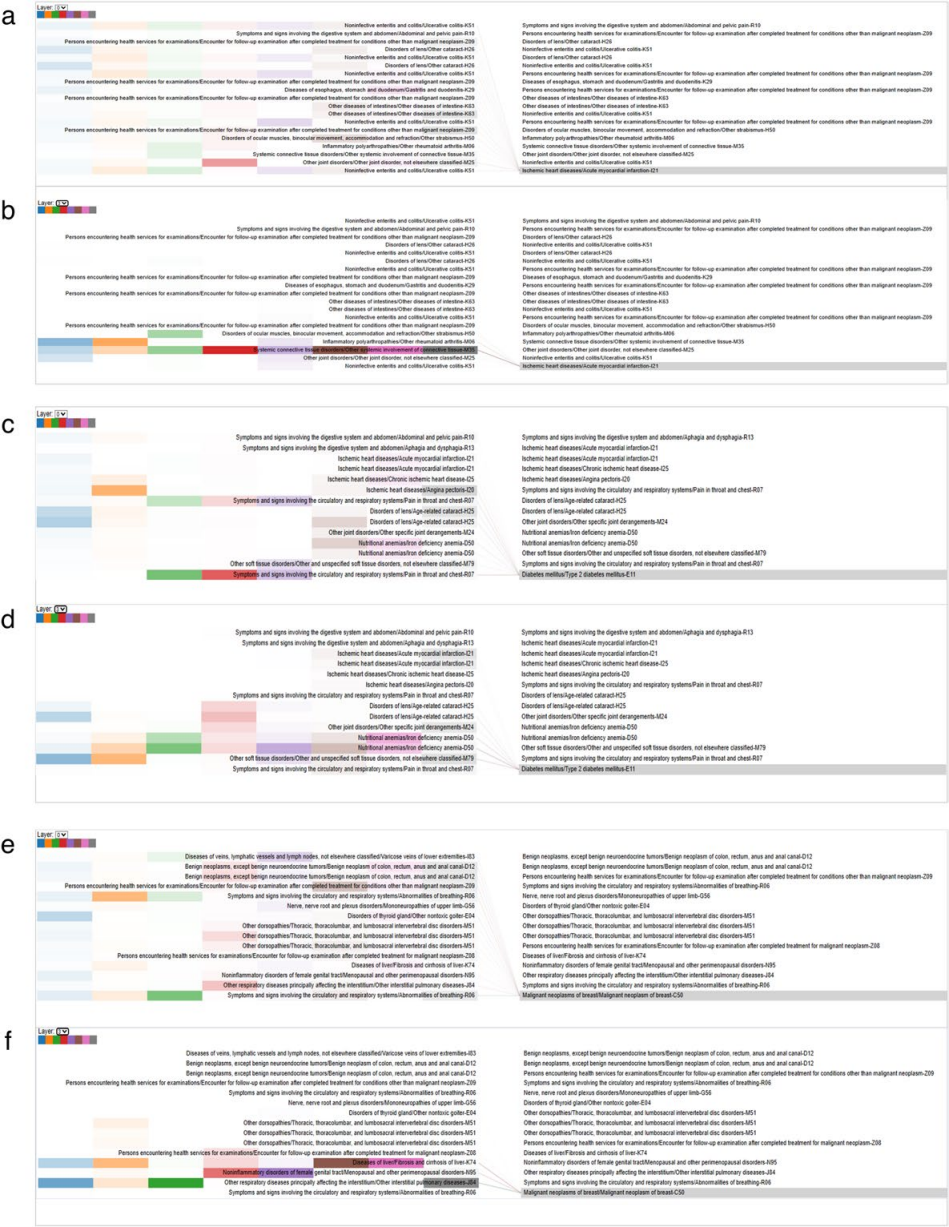
Examples of attention patterns for CAD, T2D, and BC. Connection lines from the code in the left panel to the code in the right panel indicate attention patterns of the Med-Causal-Transformer model. Panels** a** and** b** represent the CAD sample’s zero and third attention layers, respectively. Panels** c** and** d** depict the zero and third attention layers of the T2D sample. Panels** e** and** f** correspond to the BC sample’s zero and third attention layers

Med-Causal-Transformer not only can improve prediction performance but also reveal meaningful disease relationships through its attention layers, providing interpretability to the information learned by the model. Below, we present three distinct examples with CAD, T2D, and BC respectively, demonstrating how preceding diseases correlate with subsequent ones based on the attention weights derived from the model.

To visualize these attention patterns, we employ the Bertviz tool [35]. Our analysis reveales that while each layer within Transformer learns similar information, there are notable differences in the attention weights across layers. Specifically, the first two layers display smaller and more inconsistent connection weights across different attention heads. In contrast, the final two layers exhibit larger and more consistent weights, suggesting that these layers are more confident in establishing disease relationships.

Figure 6 showcases three distinct attention patterns for CAD, T2D, and BC, effectively illustrating the disease relationships learned by the model. Systemic connected tissue diseases show a heavy link with CAD in Fig. 6b [40, 41]. Similarly, Fig. 6d highlights a strong consistent link between iron deficiency anemia and T2D [42–44]. Lastly, in Fig. 6f, liver cirrhosis and interstitial pulmonary diseases show a pronounced correlation with BC. These associations are well aligned with previous authoritative research [45, 46], further validating the Med-Causal-Transformer s capability to extract meaningful disease-disease interactions. This interpretability is crucial for enhancing our understanding of complex disease dynamics and for making informed decisions in clinical settings.

## Conclusions

In this study, we introduce the Med-Causal-Transformer model, a approach for extracting critical information from medical history records (MHR) to improve disease risk prediction. This model shows two key advantages: first, it can process MHR of varying lengths and convert them into formats suitable for model training; second, it employs masked attention modules to ensure that future information is not inadvertently incorporated at each time node. These features enable the extraction of high-quality features that are specifically tailored for integration into predictive models, improving prediction performance, as evidenced by the observed performance gains (see Table 1 and Fig. 5).

Building on this foundation, we develop MIDRP, a robust and scalable deep learning model designed to predict disease risk scores by integrating genetic factors, lifestyle habits, physical measure features, and the extracted MHR information. Through experimental comparisons with and without MHR data, we observe a significant information gap between MHR and other data types. This finding validates the effectiveness of MHR data and also highlights the importance of incorporating MHR into predictive models for complex diseases. We also compared MIDRP with several existing baseline models, including LDPred2, random forest, multilayer perceptron, logistic regression, AdaBoost, DiseaseCapsule, EIR, and Med-Bert. MIDRP demonstrats state-of-the-art performance, achieving AUROC scores of 0.783 for CAD, 0.841 for T2D, and 0.784 for BC.

A visualization tool is employed to showcase the attention patterns within the Transformer module, highlighting the Med-Causal-Transformer s ability to uncover complex disease interrelations. By examining three specific cases with CAD, T2D, and BC respectively, we observed certain patterns of disease relationships which are aligned with the findings from previous authoritative studies [40–46]. This alignment not only validates the capabilities of the Med-Causal-Transformer but also demonstrates the effectiveness of MHR-based information. These findings provide robust evidence that integrating disease interrelationship information can improve the accuracy of complex disease prediction.

Looking forward, we believe that MHR may still contain some untapped latent information that remains to be explored. Future research could focus on constructing complex disease relation networks based on MHR and investigating the prediction of diseases with limited training samples using these disease correlations. Such directions could deepen our understanding of disease mechanisms and improve predictive accuracy in scenarios with limited data availability. Despite the wealth of medical data, such as high-resolution medical images, methylation, transcriptome, etc, integrating multiple modalities of health-related data to deduce clinically relevant conclusions remains challenging. Therefore, the development of multimodal models capable of processing various data types for disease prediction holds significant promise for the future.

## Supplementary Information

Below is the link to the electronic supplementary material.

Supplementary file 1 (docx 11 KB)

Supplementary file 2 (xlsx 3751 KB)

Supplementary file 3 (xlsx 3167 KB)

Supplementary file 4 (xlsx 45 KB)

Supplementary file 5 (docx 15 KB)

## Data Availability

Our code is publicly available at https://github.com/ericcombiolab/MIDRP. The data underlying this article will be shared on reasonable request to the corresponding author.
