# Supplementary material for: Causal Transformer for Learning Embeddings from Structured Medical History Records and Multi-Source Data Integration for Complex Disease Risk Prediction
Source: Interdiscip Sci. 2025 Sep 17;18(2):614–27. doi: 10.1007/s12539-025-00749-9 (PMC13219203; doi:10.1007/s12539-025-00749-9)
Supplement: Supplementary file 1 — (docx 11 KB) [file 12539_2025_749_MOESM1_ESM.docx]

**File name: Supplementary Data A**

Description: The Single Nucleotide Polymorphisms (SNPs) that were used to calculate Mendelian randomization for various exposure variants (lifestyles and physical measures).

**File name: Supplementary Data B**

Description: The SNPs filtered out by the Phenoscanner tool for various exposure variants (lifestyles and physical measures).

**File name: Supplementary Data C**

Description: The Mendelian randomization scores for CAD, T2D, and BC.
