# Supplementary material for: Causal Transformer for Learning Embeddings from Structured Medical History Records and Multi-Source Data Integration for Complex Disease Risk Prediction
Source: Interdiscip Sci. 2025 Sep 17;18(2):614–27. doi: 10.1007/s12539-025-00749-9 (PMC13219203; doi:10.1007/s12539-025-00749-9)
Supplement: Supplementary file 5 — (docx 15 KB) [file 12539_2025_749_MOESM5_ESM.docx]

**Supplementary Information for**

**Causal Transformer for Learning Embeddings from Structured Medical History Records and Multi-Source Data Integration for Complex Disease Risk Prediction**

Zeming Li^1^, Yu Xu^1^, Debajyoti Chowdhury^2^, Hip Fung Yip^2^, Chonghao Wang^2^, Lu Zhang^1,3*^

^1^ Department of Computer Science, Hong Kong Baptist University, Kong Kong SAR, 999077, China.

^2^ School of Chinese Medicine, Hong Kong Baptist University, Kong Kong SAR, 999077, China.

^3^ Institute of Systems Medicine and Health Science, Hong Kong Baptist University, Kong Kong SAR, 999077, China.

^*^ Corresponding author. E-mail: [ericluzhang@hkbu.edu.hk;](mailto:ericluzhang@hkbu.edu.hk;)

Contributing authors. E-mail(s): [cszmli@comp.hkbu.edu.hk;](mailto:cszmli@comp.hkbu.edu.hk;) csyuxu@comp.hkbu.edu.hk; debajyoti.bionova@gmail.com; 20481926@life.hkbu.edu.hk; cschwang@comp.hkbu.edu.hk;

**Supplementary Tables**

**Supplementary Table 1. Model Categories and Their Characteristics.**

| **Model Category** | **Representative Methods** | **Input Data Types** | **Data Representation** | **Feature Selection** |
| --- | --- | --- | --- | --- |
|  |  |  |  |  |
| genetic variant | LDPred2 | SNPs | (0,1,2) allele counts | GWAS significant variants |
| PCA-genetic variant | RF, MLP, LR, AdaBoost, DiseaseCapsule | gene-level SNP PCA scores | continuous PCA components | gene-based aggregation |
| genetic + non-genetic | EIR | SNPs (*P*-value<0.05), lifestyles | (0,1,2) allele counts + continuous/discrete | commonly used features |
| MHR-based | Med-BERT | MHR (ICD codes) | sequential event codes | first-level diagnosis |
| integrated | MIDRP | SNPs, lifestyles, physical measures, MHR (ICD codes) | mixed representation | MR + causal selection + first-level diagnosis |
